# P-805. Factors associated with mortality and morbidity of patients with methicillin-resistant *Staphylococcus aureus* bacteremia in a regional hospital

**DOI:** 10.1093/ofid/ofae631.997

**Published:** 2025-01-29

**Authors:** Annabel K T Choy, Tommy H C Tang, T C Wu, W S Leung

**Affiliations:** Kwong Wah Hospital, Hong Kong, Hong Kong, Hong Kong; Queen Elizabeth Hospital, Hong Kong SAR, China, Hong Kong, Not Applicable, Hong Kong; Queen Elizabeth Hospital, Hong Kong SAR, China, Not Applicable, Hong Kong; Kwong Wah Hospital, Hong Kong SAR, China, Not Applicable, Hong Kong

## Abstract

**Background:**

Methicillin-resistant *Staphylococcus aureus* (MRSA) bacteremia is a critical health issue. This retrospective study aimed at identifying factors associated with adverse clinical outcomes in MRSA bacteremic patients admitted to Kwong Wah Hospital, an 800-bed regional hospital in Hong Kong, China. It was approved by the Kowloon Central/Kowloon East Cluster Research Ethics Committee, Hospital Authority (KC/KE-23-0107/ER-4).

**Figure:**
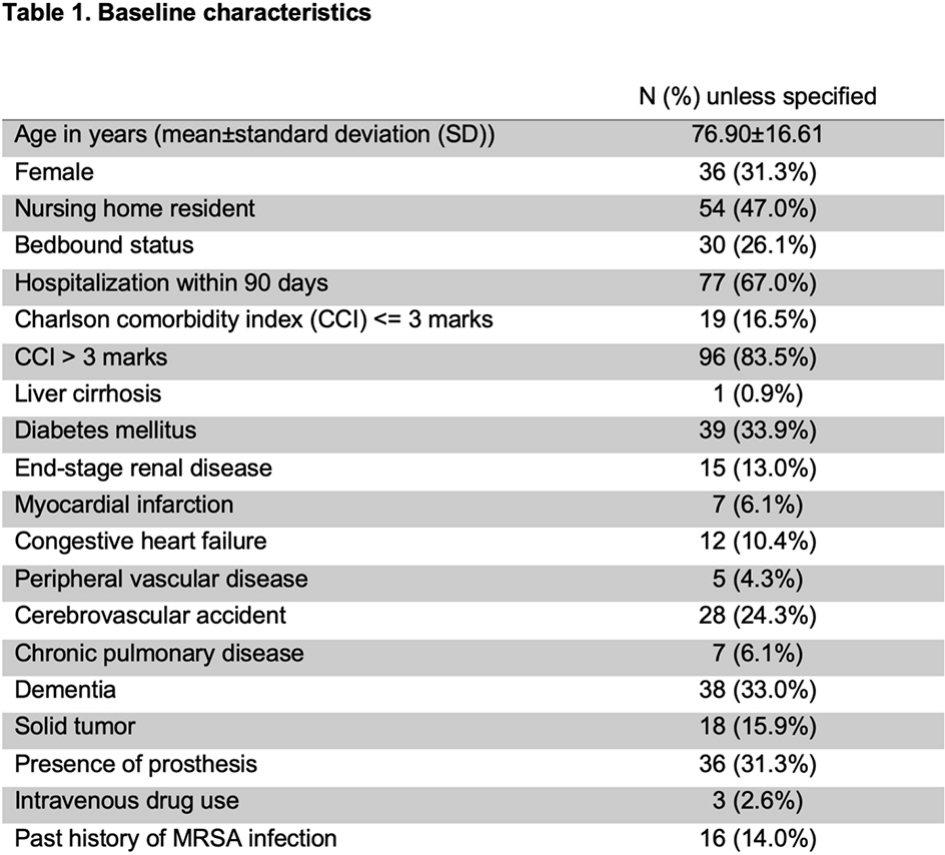

**Methods:**

Patients aged 18 years or above with MRSA bacteremia between 2020 and 2022 were identified. Their demographics and clinical background were summarized in **Table 1**. The primary and secondary outcomes were 30-day all-cause mortality and intensive care unit (ICU) admission, respectively.

**Figure:**
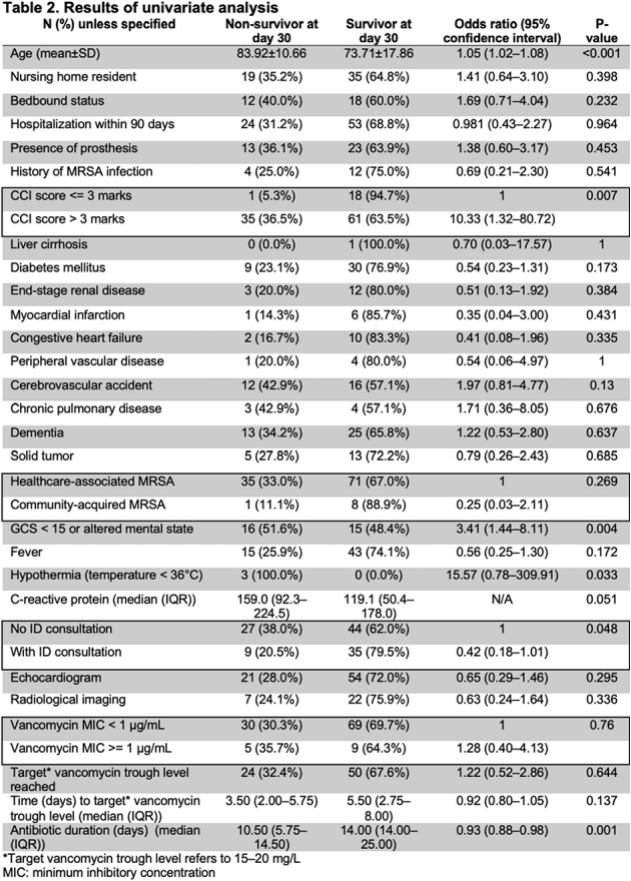

**Results:**

Of the 115 patients included, 112 (97.4%) received intravenous vancomycin as the first line antibiotic, 59 (51.3%) developed complicated bacteremia and 17 (14.8%) necessitated second line antibiotics (**Figure 1**). Thirty-six (31.3%) died within 30 days of positive blood culture for the first time and 14 (12.2%) needed ICU care.

Among the 44 (38.3%) patients attended by an infectious disease (ID) doctor, there was a higher chance to receive echocardiogram (77.3% vs 56.3%), computed tomography (52.3% vs 8.45%), surgical removal of infective foci (43.2% vs 12.7%), and numerically lower deaths (20.5% vs 38.0%) compared to those who were not.

ID consultation, along with younger age, lower Charlson comorbidity index, longer duration of antibiotics, and those without altered mental status or hypothermia, were noted to favor survival in univariate analysis (**Table 2**). By multivariate logistic regression, older age, higher C-reactive protein, altered mental states, and shorter antibiotic duration were significantly associated with mortality while ID consultation, vancomycin MIC >= 1 μg/mL, and congestive heart failure were associated with ICU admission (**Table 3**).

**Figure:**
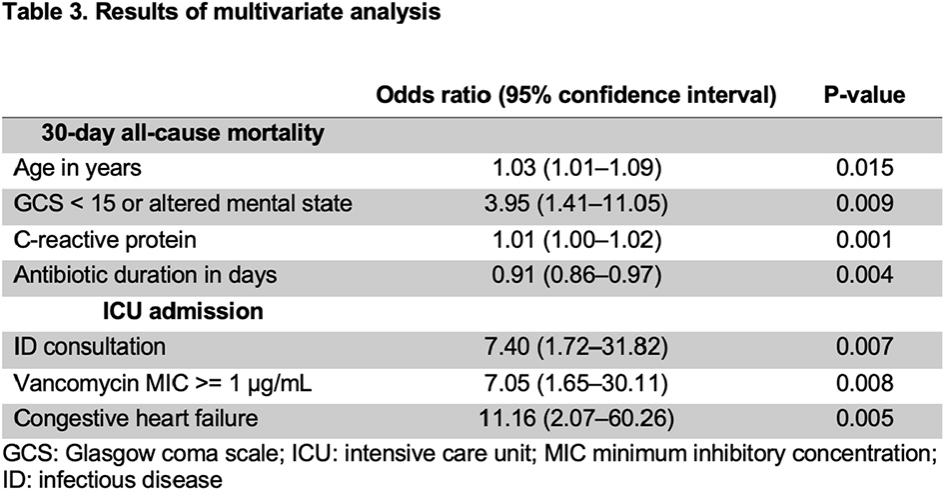

**Conclusion:**

MRSA bacteremia resulted in high mortality. ID consultation might have contributed to a greater effort in searching for surgically removable infective foci and a numerically lower death rate. Its association with ICU admission might suggest that sicker patients require greater expertise from ID doctors.

**Figure:**
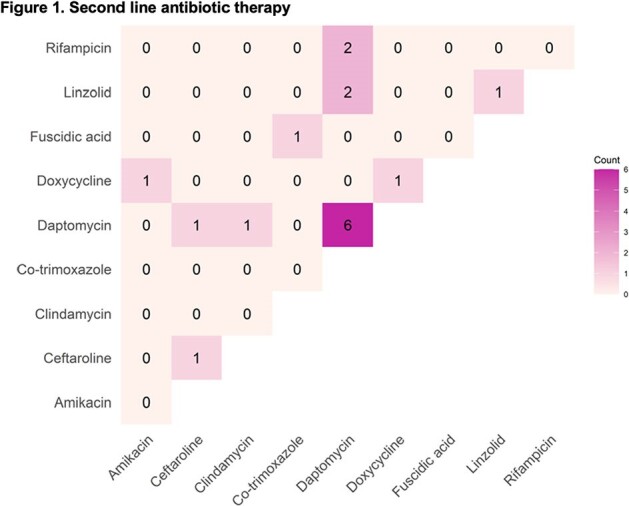

**Disclosures:**

**All Authors**: No reported disclosures

